# Identification and functional analysis of c.422_423InsT, a novel mutation of the *HNF1A* gene in a patient with diabetes

**DOI:** 10.1002/mgg3.261

**Published:** 2016-11-30

**Authors:** Jesús Miguel Magaña‐Cerino, Juan P. Luna‐Arias, María Luisa Labra‐Barrios, Bartolo Avendaño‐Borromeo, Xavier Miguel Boldo‐León, Mirian Carolina Martínez‐López

**Affiliations:** ^1^ Centro de Investigación y PosgradoLaboratorio de Diagnóstico MolecularDivisión Académica de Ciencias de la Salud (DACS)Universidad Juárez Autónoma de Tabasco (UJAT)Ave. Gregorio Méndez Magaña. No 2838‐A, Col. Tamulté de las BarrancasVillahermosaC.P. 86150México; ^2^ Departamento de Biología CelularCentro de Investigación y de Estudios Avanzados del Instituto Politécnico Nacional (CINVESTAV‐IPN)Ave. Instituto Politécnico Nacional 2508, Col. San Pedro ZacatencoCiudad de MéxicoC.P. 07360México

**Keywords:** Dominant‐negative effect, *HNF1A*, I27L, MODY3, Q141Hfs*47

## Abstract

**Background:**

*HNF1A* gene regulates liver‐specific genes, and genes that have a role in glucose metabolism, transport, and secretion of insulin. *HNF1A* gene mutations are frequently associated with type 2 diabetes. HNF1A protein has three domains: the dimerization domain, the DNA‐binding domain, and the trans‐activation domain. Some mutations in the dimerization or DNA‐binding domains have no influence on the normal allele, while others have dominant negative effects. The I27L, A98V, and S487N polymorphisms are common variants of the *HNF1A* gene; they have been found in T2D and non‐diabetic subjects.

**Methods and Results:**

We searched for mutations in the first three exons of the *HNF1A* gen in an Amerindian population of 71 diabetic patients. DNA sequencing revealed the previously reported I27L polymorphism (c.79A>C) in 53% of diabetic patients and in 67% of the control group. Thus, the I27L/L27L polymorphism might be a marker of Amerindians. In addition, we found the c.422_423InsT mutation in the *HNF1A* gene of one patient, which had not been previously reported. This mutation resulted in a frame shift of the open reading frame and a new translation stop in codon 187, leading to a truncated polypeptide of 186 amino acids (Q141Hfs*47). This novel mutation affects the DNA‐binding capacity of the mutant HNF1A protein by EMSA; its intracellular localization by fluorescence and confocal microscopy, and a dominant‐negative effect affecting the DNA‐binding capacity of the normal HNF1A by EMSA. We also studied the homology modeling structure to understand the effect of this mutation on its DNA‐binding capacity and its dominant negative effect.

**Conclusion:**

The HNF1A Q141Hfs*47 mutant polypeptide has no DNA‐binding capacity and exerts a dominant negative effect on the HNF1A protein. Therefore, it might produce severe phenotypic effects on the expression levels of a set of *β*‐cell genes. Consequently, its screening should be included in the genetic analysis of diabetic patients after more functional studies are performed.

## Introduction

The maturity onset diabetes of the young (MODY) is a monogenic form of diabetes characterized by an autosomal dominant inheritance; the onset usually happens before the 25 years of age and is characterized by an impaired insulin secretion with minimal or no defect of the insulin action (Fajans and Bell 2001). Some studies suggest that 1–2% of patients with type 2 diabetes (T2D) may in fact have MODY (Shields et al. 2010). Data available suggest that people carrying one mutated allele are born with completely normal physiological and biochemical functions of the pancreatic *β*‐cells, and diabetes will occur at some stage during adolescence (Bell and Polonsky 2001; Fajans and Bell 2001). Penetrance of diabetes in patients with mutations in MODY is quite high (more than 95% by the age of 55 years) (Frayling et al. 2001; Murphy et al. 2008). Recent studies have demonstrated heterozygous mutations in genes encoding 11 forms of MODY, including the hepatocyte nuclear factor‐4*α* encoding the gene (*HNF4A*)(MODY 1), the glucokinase gene or *GCK* (MODY 2), the hepatocyte nuclear factor‐1*α* that encodes *HNF1A* (MODY 3), the pancreas/duodenum homeobox protein 1 (*PDX1,* also known as *IPF‐1*) (MODY 4), the hepatocyte nuclear factor‐1*β* encoding the gene *HNF1B* (MODY 5), the neurogenic differentiation 1 that encodes the gene (*NEUROD1*)(MODY 6), the Kruppel‐like factor 11 (*KLF11*) (MODY 7), the carboxyl‐ester lipase encoding the gene (*CEL*) (MODY 8), the paired box gene 4 (*PAX4*) (MODY 9), insulin gene (*INS*)(MODY 10), the tyrosine kinase B‐lymphocyte specific gene (*BLK*) (MODY 11), the potassium voltage‐gated channel subfamily J member 11 (*KCNJ11* gene) (MODY13), and the adapter protein containing PH domain, PTB domain and leucine zipper motif 1, also known as DCC‐interacting protein 13‐*α* encoded by the *APPL1* gene (MODY14). Those cases of as yet unknown genetic derangement have been classified as MODYX (Online Mendelian Inheritance in Man [OMIM], MIM entry 606391).

Mutations in the *HNF1A* gene (OMIM 142410) are the most common cause of MODY (Shields et al. 2010). Furthermore, three frequent polymorphisms (I27L, A98V, and S487N) have been described in patients with T2D (Holmkvist et al. 2006; Giuffrida et al. 2009; Bonatto et al. 2012). Characteristics of MODY carriers are: (1) Early age of diabetes onset, (2) overweight or obesity are rarely associated, (3) the levels of insulin are usually within the normal range, though inappropriately low for the degree of hyperglycemia, (4) family history of diabetes in at least three generations, showing an autosomal dominant mode of inheritance, and (5) show non‐insulin dependence (Klupa et al. 2002; Vaxillaire and Froguel 2008). The HNF1A encoded protein is a transcription factor expressed in liver, kidney, intestine, stomach, and pancreas (Harries et al. 2009; Bae et al. 2010). It regulates liver‐specific genes and genes involved in glucose metabolism, transport, and insulin secretion (Wu et al. 1994; Okita et al. 1999; Cerf 2006). The HNF1A protein has three functional domains: (1) the N‐terminal dimerization domain (DD), which corresponds to amino acids 1–32. (2) the DNA‐binding region (DBD) (amino acids 91–279) divided in two homeodomains, POU_S_ (amino acids 91–181) and POU_H_ (amino acids 203–279), linked by a structurally disordered region encompassing amino acids 182 to 200. This region also contains a nuclear localization signal (NLS) (amino acids 197–205) (Chi et al. 2002). The sequence of the POUs – HNF1A domain, highly differs from other POUs domains in other polypeptides; however, its structural properties are similar (Chi et al. 2002). (3) The C‐terminal domain is the one containing the transactivation domain (TAD) (amino acids 281–631) (Vaxillaire et al. 1999; Bjørkhaug et al. 2005).

The HNF1A protein binds to DNA as a homodimer or heterodimer with the structurally related HNF1B protein (Rose et al. 2000). The DCoH polypeptide has been identified as a dimerization cofactor of the HNF1A protein, which displays a restricted tissue distribution and does not bind to DNA, but selectively stabilizes HNF1A dimmers (Mendel et al. 1991; Rho et al. 2010). Also, it has been shown that the dimerization motif of the HNF1A protein forms an intermolecular 4‐helix bundle (Hua et al. 2000) which can be destabilized by a subset of MODY‐associated mutations. Thus, impaired dimerization of the beta‐cell transcription factor provides a molecular mechanism of metabolic deregulation in T2D (Hua et al. 2000).

Heterozygous knockout mice that lack in one copy of the *HNF1A* have a normal phenotype, whereas MODY patients have a heterozygous mutation and fully express the diabetes phenotype (Pontoglio et al. 1998; Pontoglio 2000). This observation suggests that these mutations might have a dominant negative effect or haploinsufficiency (Wang et al. 1998; Hagenfeldt‐Johansson et al. 2001). Experimental data have shown that only mutations in the transactivation domain of the HNF1A protein have a dominant negative effect over the normal HNF1A transactivation potential. Mutations located in either the dimerization domain or in the DNA‐binding domain do not interfere with the activity of the normal allele (Vaxillaire et al. 1999). However, the mutation R272C is an exception, which causes the loss of DNA‐binding activity of the HNF1A protein in vitro, and exerts a dominant negative effect in vivo (Yoshiuchi et al. 1999).

In this work we found the I27L as the most frequent polymorphism in the studied population (patients and controls). Outstandingly, a new mutation of the *HNF1A* gene was found in a patient with T2D, the c.422_423InsT mutation. This mutation caused a frame shift and consequently a truncated polypeptide of 186 amino acids in length. We determined that this novel mutation affects the DNA‐binding capacity of the mutant HNF1A polypeptide and has a dominant negative effect over the DNA‐binding capacity of the normal HNF1A protein in vitro.

## Materials and Methods

### Patients and controls

Seventy‐one patients (45 women, 26 men) diagnosed with diabetes according to the NOM‐015‐SSA2‐2010 criteria (Mexico) participated in this study. The mean age at the time of their diagnosis was 34.15 ± 5.73 years. Sixteen patients (22.54%) had no family history of diabetes, while 55 (77.46%) had a family record of diabetes in at least one first‐degree relative. The control group consisted of healthy individuals without a history of diabetes or any other family disease (*n* = 34; 24 women, 10 men), and the mean age was 21.85 ± 0.5 years. All the members of this group were confirmed as non‐diabetic by the oral tolerance glucose test (OTG) with an overload of 75 g anhydride glucose; an immediate fasting plasma glucose concentration <110 mg/dL (6.1 mmol/L), or after 2 h <200 mg/dL (11.1 mmol/L) were considered as normal. Biochemical measurements were performed with a Metrolab 2300 Plus Analyzer (Buenos Aires, Argentina): Glucose, total cholesterol, triglycerides, HDL, and LDL levels were determined by colorimetry, using regents specified by the supplier (Wiener Lab, Rosario, Argentina). The insulin concentration was measured by chemiluminescence with an Inmulite 1000 Immunoassay System (Siemens, Munich, Germany). HbA1c was measured using the Glycohemoglobin Unitest (Eagle diagnostics, Desoto, TX, USA). The homeostatic model assessment (HOMA) was estimated using the HOMA2 calculator (http://www.dtu.ox.ac.uk/homacalculator/). An informed consent was obtained from each subject. The Ethics Committees of the Academic Division of Health Sciences at the Juarez Autonomous University of Tabasco (División Académica de Ciencias de la Salud, Universidad Juárez Autónoma de Tabasco, in Spanish), as well as of The Dr. Juan Graham Casasús Hospital (both institutions in Villahermosa, Tabasco) approved this project.

### Amplification and sequencing of the *HNF1A* gene exons

Genomic DNA from whole blood was isolated with the Wizard Genomic DNA Purification kit (Promega Madison, WI, USA). First three *HNF1A* (GenBankID M57732) exons were amplified because they contain the most frequent mutations in the American Caucasian population (Urhammer et al.^1998^; Baier et al.^2000^; Klupa et al. 2002; Furuzawa et al.^2008^). The following oligonucleotides were used: Exon 1, M3E1F (5′‐TGAGCCAGCTGCAGACGGAG‐3′) and M3E1R (5′‐CTCTAGGCTCTCCTGGGAGC‐3′) (Tm 64.3°C, 356 bp amplicon size); Exon 2, M3E2F (5′‐CAGCCCTTGCTGAGCAGATC‐3′) and M3E2R (5′‐GAGTTAGGGGAGAGTCCTGG‐3′) (Tm 61.2°C, 299 bp amplicon size); Exon 3, M3E3R (5′‐GACGAGGGAAGGTGAGAGTG‐3′) and M3E2R (5′‐CCCAGACCAAACCAGCACTG‐3′) (Tm 62.3°C, 282 bp amplicon size). The amplicons were amplified by PCR in an Eppendorf Mastercycler Personal System; the electrophoresis was performed in 1% agarose gels and observed in a Gel Doc EZ Imager (Bio‐Rad, Hercules, CA, USA), then it was purified with the Pure Link Quick Gel Extraction Kit (Invitrogen Waltham, MA, USA), and sequenced with the Big Dye Terminator v3.1 Cycle Sequencing kit and an ABI PRISM 3100 Genetic Analyzer (Applied Biosystems Austin, TX, USA).

### Site‐directed mutagenesis of the *HNF1A* gene

The novel c.422_423InsT mutation was introduced into the *HNF1A* gene using the pHNF1A3.1/HisC plasmid (a kind gift from Dr. Lise Bjørkhaug Gundersen, University of Bergen, Norway), the Quick Change II Site‐Directed Mutagenesis kit (Agilent Technologies Santa Clara, CA, USA), and the oligonucleotides 422_423insT_HNF1AF (5′‐CTGGCCTCAACCA**T**GTCCCACCTGTCC‐3′) and 422_423insT_HNF1AR (5′‐GGACAGGTGGGAC**A**TGGTTGAGGCCAG‐3′) (the mutated base is underlined and bolded). The plasmid pHNF1A3.1/HisC_c.422_423InsT was purified with the QIAprep Spin Miniprep kit (Qiagen Germantown, MD, USA) and the mutation was confirmed by sequencing.

### In vitro HNF1A and HNF1A 422_423insT protein expression

Normal and Q141Hfs*47 mutant HNF1A polypeptides, either unlabeled or ^35^S‐Methionine‐labeled, were synthesized using the TNT T7 Quick Coupled Transcription/Translation kit (Promega Madison, WI, USA) using the plasmids carrying the normal and the 422_423insT mutant versions of the *HNF1A* gene, following the manufacturer's instructions. Briefly, 2 μg of each plasmid were used for the synthesis of recombinant polypeptides in a final volume of 50 μL and incubated at 30°C for 90 min. For the radioactive reactions, we used 1 μL (10.2 μCi) of L‐^35^S‐Methionine (10.2 mCi/mL; specific activity 1175.0 Ci/mmol, Perkin Elmer, Waltham, MA, USA); after labeling, 1 μL of 1 mm methionine was added and incubated for 30 more minutes at the same temperature. Then, the samples were kept at −70°C until use. As a control, we performed a synthesis reaction using the pcDNA3.1 His C empty vector. To visualize the radio‐labeled proteins, 5 μL of each reaction were mixed with 5 μL of 2× Laemmli sample buffer and heated at 85°C for 10 min as recommended, then they were electrophoresed in a 10% SDS‐PAGE gel. Next, the proteins were stained with Coomassie blue, distained with methanol/acetic acid/deionized water (50/10/40, v/v/v), rinsed in 10 mL of EN^3^HANCE (Perkin Elmer, Waltham, MA, USA), kept in agitation for 30 min, covered with saran wrap, exposed to an Amersham Hyperfilm ECL (GE Healthcare, Chicago, IL, USA) for 1 month at −80°C, and finally developed with KODAK GBX developer.

### Electrophoretic mobility shift assays (EMSA)

Five micro liters of each in vitro transcription/translation reactions were incubated in a mixture containing 1× DNA mix (1 mm spermidine, 1 mm MgCl_2_), 1 μg poly (dG/dC)·poly (dG‐dC) (Amersham Pharmacia Biotech, GE Healthcare, Chicago, IL, USA) and 1× binding buffer (12 mm Hepes pH 7.9, 60 mm KCl, 10% glycerol, 1 mm EDTA, 1 mm MgCl_2_, 1 mm DTT) for 30 min at 4°C in a final volume of 19 μL. Then, 1 μL of dsDNA‐labeled probe (40,000 cpm, 1.29 nm), corresponding to the HNF1A‐binding site (5′‐CTCAGTAAAGATTAACCAT‐3′) from the *GLUT2* promoter (Ban et al. 2002; Cervin et al. 2002), was added to each mixture and the reactions were incubated during 15 min at 4°C. The probe was labeled with [*γ*‐^32^P]‐ATP (3000 Ci/mmol, Perkin Elmer, Waltham, MA, USA) and T4 polynucleotide kinase (New England BioLabs, Ipswich, MA, USA). In competition experiments, 300‐fold molar excess of either unlabeled probe or *Entamoeba histolytica* TATA‐box (5′‐AATTCTCTATTTAAAGAGAATT‐3′) (Castañon‐Sanchez et al. 2010) as unspecific competitors were added to the mixture reaction 30 min before adding the labeled probe. For evaluating the effect of the mutant HNF1A Q141Hfs*47 on the HNF1A binding activity, increasing quantities of the in vitro translated HNF1A Q141Hfs*47 (0.5, 1, 2, and 4‐fold volume excess) were added, maintaining the quantity of HNF1A fixed, and then assayed with EMSA. The final reaction volume was increased up to 50 μL and incubated with labeled‐probe (40,000 cpm, 0.52 nm) for 30 min at 4°C. The reactions were electrophoresed in 6% non‐denaturing PAGE gels using 0.5× TBE (44.5 mm Trizma base, 44.5 mm boric acid, 1 mm EDTA), then they were vacuum‐dried in a 583 Model Gel Dryer (Bio‐Rad, Hercules, CA, USA), subsequently they were exposed to an Imaging Screen K (Bio‐Rad), and scanned in a Molecular Imager FX (Phosphor Imager) apparatus (Bio‐Rad, Hercules, CA, USA). All the experiments were performed at least twice. Complexes formed by the HNF1A protein and [^32^P]‐labeled dsDNA probe were quantified by densitometry, measuring the pixels in each band and subtracting the corresponding background for each lane using the Quantity One software version 4.6.2 (Bio‐Rad, Hercules, CA, USA), and normalized against free probe to correct the total cpm loaded in each lane.

### In silico analysis

The prediction of the HNF1A protein structure and function was done using physical and comparative straightforward considerations in the PolyPhen‐2 web server (http://genetics.bwh.harvard.edu/pph2/). The protein structure of the mutant HNF1A Q141Hfs*47 was performed by homology modeling with the Modeller program at the ModWeb Comparative Modeling Server (version SVN.r1368M, https://modbase.compbio.ucsf.edu/scgi/modweb.cgi) selecting the best, longest scoring model, and slow modeling processes. The Query protein (residues 87–164) was modeled using as a template the corresponding region of the HNF1A bound to DNA (RCSB Protein Data Bank ID 1IC8). The structure of the HNF1A with no DNA bound was also calculated in ModWeb to determine its electrostatic potential (EP) on surface.

The protein structures were visualized with UCSF Chimera (version 1.8, build 38824, http://www.cgl.ucsf.edu/index.html) and the Molegro Molecular Visualizer (MMV) (version 2.5, CLC bio) programs. A comparison of the structures was done with the Match Maker module of Chimera program, using the Needleman–Wunsch algorithm and BLOSUM62 matrix for best‐aligning pair of chains. A quality model evaluation was performed with PROCHECK and VERIFY_3D programs at the Structural and Analysis Verification Server (SAVES) from the National Institutes of Health MBI Laboratory for Structural Genomics and Proteomics at the University of California, Los Angeles (http://nihserver.mbi.ucla.edu/). The HNF1A Q141Hfs*47 homology model was deposited in The Protein Model Database from CASPUR and the Bio‐computing group of the Department of Biochemical Sciences of the University of Rome “La Sapienza” (https://bioinformatics.cineca.it/PMDB/main.php), with the identifier PM0079278. To determine the EP of the normal and mutant HNF1A molecules, first the PDB files were converted to a PQR format using the PDB2PQR version 1.8 server at the National Biomedical Computation Resource web site (http://nbcr.ucsd.edu/) employing the default parameters in the program (force field, PARSE; optimization of hydrogen bonding network). Then, the EP of molecular models was determined with the Adaptive Poisson‐Boltzmann Solver (APBS) software following the web link mentioned above. Finally, the EP on protein surface was visualized with Chimera (setting values between −5 (red) to +5 (blue) Kcal/mole).

### Intracellular localization of HNF1A and HNF1A Q141Hfs*47 in COS‐7 cells

The plasmids pHNF1A3.1/His C and pHNF1A3.1/HisC_c.422_423InsT were used as templates to amplify the *HNF1A*gene (1910 bp) and the mutant *HNF1A c.422_423InsT* (584 bp), using the oligonucleotides: forward HNF1AWT/MUT_EcoRI_pEGFPN1_FW for both amplicons, 5′‐CCGGAATTCATGGTTTCTAAACTGAGCCAGC‐3′; HNF1AWT_BamHI_pEGFPN1_AS; the reverse oligonucleotide for full‐length *HNF1A* gene was 5′‐CGCGGATCCTGGGAGGAAGAGGCCATC‐3′; the reverse primer was HNF1AMut_BamHI_pEGFPN1_AS for *HNF1A* 422_423InsT was 5′‐CGCGGATCCCGGAATTCATCAGCCCTCC‐3′ (the *Eco*RI and *Bam*HI restriction sites appear underlined). The amplifications were done with Phusion High‐Fidelity DNA polymerase (New England BioLabs), the products were purified and cloned into a TOPO TA vector (Invitrogen), and subcloned into pEGFP‐N1 (a kind gift from Dr. Guadalupe Reyes, CINVESTAV‐IPN) with the Liga Fast Rapid DNA Ligation System (Promega Madison, WI, USA) to generate the plasmids pEGFP‐N1/HNF1A and pEGFP‐N1/HNF1A_c.422_423InsT, which were purified (Qiagen Plasmid Midi kit, Germantown, MD, USA) and sequenced.

For transfection, 100,000 COS‐7 cells were grown per well in a 24‐well plate (Corning) in a DMEM culture medium (37°C, 24 h in 5% CO_2_), they were transfected (5 μg of each plasmid) with Lipofectamine 2000 (Invitrogen) and incubated for 72 h. The total RNA was isolated with Trizol (Invitrogen). Then, cDNA synthesis was performed with the Proto Script II Reverse Transcriptase (New England BioLabs). The *HNF1A‐EGFP* mRNA expression was verified by PCR using the oligonucleotides HNF1A‐EGFP141‐FW (5′‐ATCCAACCACAGCGTCATCG‐3′) and EGFP‐REV (5′‐CCGTCCAGCTCGACCAG‐3′); the expression of the *HNF1A c.422_423InsT‐EGFP* was done with the latter oligonucleotide and the *HNF1A* 422_423InsT‐EGFP‐FW (5′‐GTCGATACCACTGGCCTCAA‐3′). The expected amplicon sizes were 141 bp and 242 bp for *HNF1A‐EGFP* and *HNF1A 422_423InsT‐EGFP*, respectively. The *β*2‐microglobulin (Gen Bank NM_004048.2, 294 bp amplicon) gene expression was used as a RT‐PCR control using the *β*2M‐FW (5′‐ATGTCTCGCTCCGTGGCCTTAGCT‐3′) and *β*2M‐REV (5′‐ATACTCATCTTTTTCAGTGGGGGT‐3′) oligonucleotides. Before microscope observation, the transfected cells were stained with 5 μg/mL of Hoechst 33342 (Molecular Probes, Waltham, MA, USA), mounted on a glass slide and immediately observed through a Leica TCP‐SP8 confocal microscope using a 63× objective. In total, 100 cells were examined per each transfection performed, and three replicates.

### Statistical analysis

The data analysis was carried out using the PAWS Statistics 18 package (SPSS Inc., Chicago, IL, USA). One‐way anova test was used to compare clinical and laboratory data. The data were expressed as means ± SD. *P* values <0.05 (two tailed) were considered as significant. The *χ* ^2^ was used to observe differences between carriers of mutant and normal alleles in the diabetic patients and controls.

## Results

### Clinical report

The metabolic differences found between the control and the diabetic groups were significant (*P* < 0.05) when comparing the levels of glucose, cholesterol, triglycerides, LDL‐cholesterol, and HOMA‐B (Table 1). Insulin levels were higher in the diabetic group due to insulin resistance as a result of high glucose levels in blood. HOMA‐B was low in comparison with control group. No significant differences in HDL levels were found when comparing both groups. Diabetic patients showed complications 10 years after the diabetes diagnosis: 13 patients (18.55%) presented retinopathy, 8 (11.4%) nephropathy, 22 (48.5%) hypertension, and only 22 (21.6%) had no complications. Only one patient (D16) presented retinopathy and diabetic nephropathy. DNA changes in the *HNF1A* gene were identified in 38 diabetic patients (53.52%) and in 23 controls (67.65%). The I27L/L27L polymorphism (substitution c.79A>C in exon1) was the most common found in both groups (Table 1) and has been previously reported in several MODY3 families (Chiu et al. 2000; Babaya et al.^2003^).

**Table 1:** Clinic and genetic characteristics of diabetic patients and controls

	Type 2 diabetic patients (*n* = 71)	Controls (*n* = 34)	*P*‐value
Average age (years)	47.11 ± 1.30	21.85 ± 0.503	–
Males/Females	26/45	10/24	–
Family history of diabetes: Yes/No	77.46%/22.54%	0% Yes	–
Glucose (mmol/L)	11.27 ± 0.56	4.85 ± 0.18	5.62 × 10^−12^ a
Total cholesterol (mmol/L)	5.62 ± 0.26	3.82 ± 0.13	1.05 × 10^−5^ a
Triglycerides (mmol/L)	3.52 ± 0.54	01.21 ± 0.14	0.004 a
HDL‐cholesterol (mmol/L)	1.28 ± 0.04	1.22 ± 0.04	0.389
LDL‐cholesterol (mmol/L)	2.91 ± 0.13	2.09 ± 0.14	2.18 × 10^−4^ a
Insulin (μU/mL)	8.38 ± 0.74	6.21 ± 0.74	0.072
HOMA‐B (%)	34.11 ± 3.89	93.96 ± 11.25	3.8 × 10^−9^ a
HNF‐1*α* mutations/WT			
I27L/L27L	38/33	23/11	0.170
Q141Hfs*47/WT	1/70	0/34	–

Values are presented as means ± SEM; HOMA, homeostasis model assessment; HDL, high‐density lipoprotein; LDL, low‐density lipoprotein.

a Statistically significant.

The patient D16 (40‐year‐old male) had a frame shift mutation (c.422_423insT) in the *HNF1A* exon 2. He presented 142 mg/dL (7.88 mmol/L) of glucose, 237 mg/dL (6.16 mmol/L) of total cholesterol, 501 mg/dL (5.71 mmol/L) of triglycerides, 66 mg/dL (1.71 mmol/L) of HDL, 100 mg/dL (2.59 mmol/L) of LDL, 4.65% (4.8 mmol/L) of HbA1c; 7.7 μU/mL of insulin, and 38.9% of HOMA‐B. He declared that there were non‐diabetic members in his family. He presented hyperlipidemia and hyperglycemia; his *β* cells were still functioning and producing insulin, and his glucose levels in blood were close to normal values. However, his family was not included in this study because they were not localized.

### In silico analysis of the *HNF1A c.422_423insT* gene mutation

The novel *HNF1A c.422_423insT* mutation is an insertion of a deoxythymidine between nucleotides 422 and 423 (Gen Bank ID NM_000545.5), which causes a change of glutamine to histidine at codon 141 and a subsequent stop codon at position 187 (Q141Hfs*47) (Gen Bank ID NP_000536.5). The mutation was located in the DBD and was found to be a probably damaging mutation by the PolyPhen‐2 program (HumVar scores: 1.0). Conservation analyses have suggested that Q141 is highly conserved among species and is located in a highly conserved region of the HNF1A protein (Ryffel 2001).

**Figure 1:**
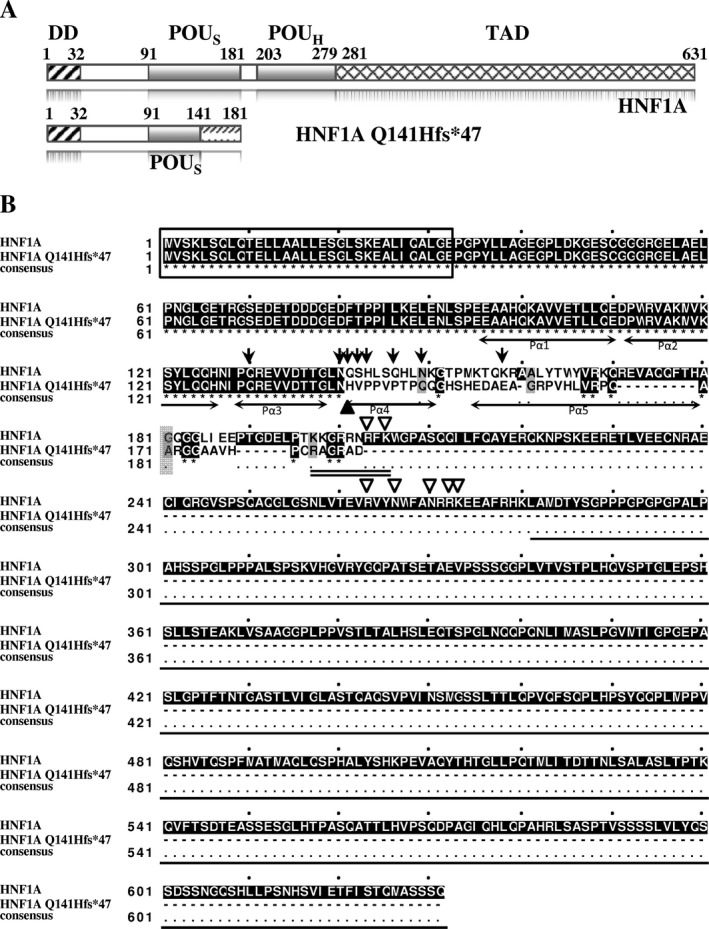
Comparison of the amino acid sequences between the HNF1A and the HNF1A Q141fs*47 polypeptides. (A) Scheme of HNF1A and HNF1A Q141fs*47 proteins showing the dimerization domain (DD, amino acids 1–32), POU domains (POU_S_, amino acids 91–181 and POU_H_, amino acids 203–279) and the transactivation domain (TAD, residues 281–631). The HNF1A Q141fs*47 polypeptide only contains the dimerization domain and a fragment of the POU_S_ domain (residues 91–140). (B) Alignment of HNF1A and HNF1A Q141fs*47 polypeptides with Clustal Omega; the identical amino acids are in black boxes and have an asterisk below; the conserved amino acids are in gray boxes; DD is indicated by a rectangular frame; amino acids of the POU_S_ domain interacting with DNA in HNF1A polypeptide are indicated with arrows, whereas residues of the POU_H_ domain that interact with DNA are indicated with empty arrowheads; H141 in the mutant polypeptide is indicated with a black arrowhead; *α*‐helixes (P*α*1 to P*α*5) of the POU_S_ domain are indicated with double‐headed arrows below the sequences; the normal NLS is double‐underlined.

The mutated HNF1A Q141Hfs*47 protein has the first 50 residues of POU_S_ domain identical to the normal protein, but the following 31 residues are only 16% identical and 25% similar to the corresponding region in HNF1A POU_S_ domain (Fig. 1). Remarkably, this mutation affected the DNA‐interacting residues Q141, S142, H143, Q146, N149, and K158 of the normal protein (Chi et al. 2002), which were changed to H141, V142, P143, P146, Q149, and E158 in HNF1A Q141Hfs*47. Furthermore, the mutated polypeptide lacks POU_H_ and TAD; consequently, its DNA‐binding activity might be severely affected. Finally, we considered that the nuclear localization sequence (NLS) could also be affected because the number of positively charged residues was reduced from six in the normal HNF1A (Chi et al. 2002) to just two in the mutated HNF1A Q141Hfs*47 (Fig. 1). Thus, the translocation to the nucleus might be compromised as well.

### The DNA‐binding activity of HNF1A Q141Hfs*47 was severely affected

**Figure 2:**
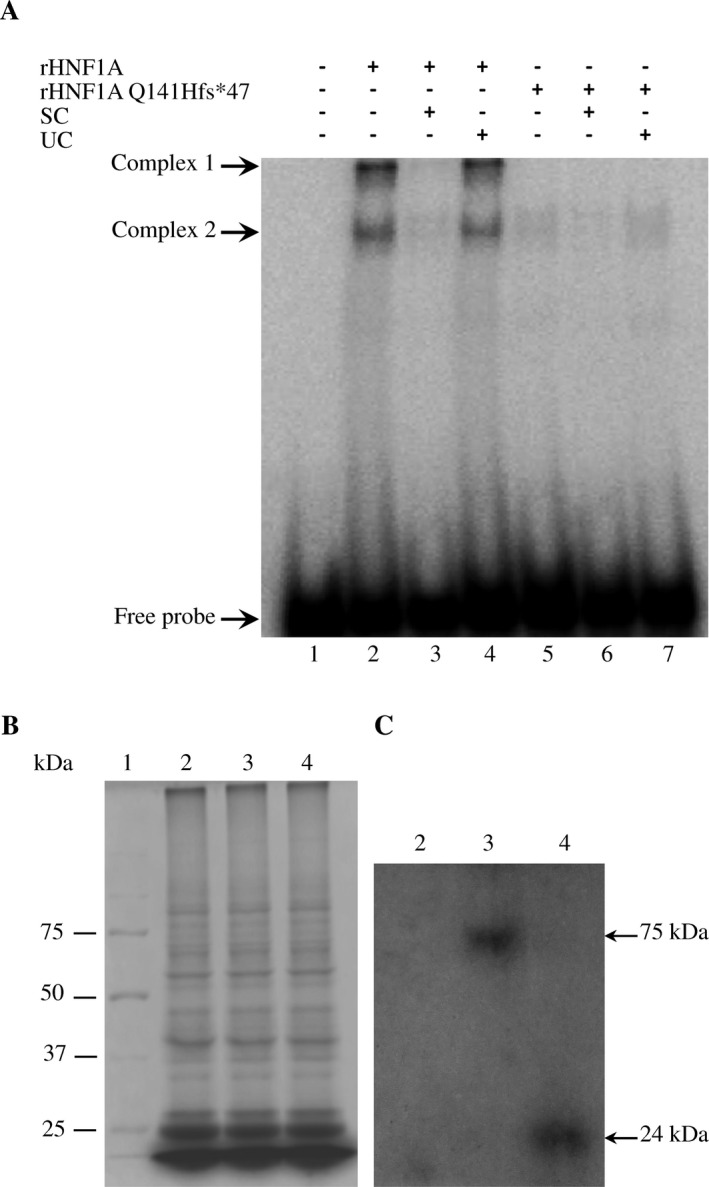
The HNF1A Q141fs*47 polypeptide lacked in DNA‐binding activity in vitro. (A) DNA‐binding activity of HNF1A and HNF1A Q141fs*47 polypeptides were assayed by EMSA. rHNF1A and rHNF1A Q141fs*47 proteins were expressed in vitro in a coupled transcription/translation system as described. Five μL of each reaction mixture were used in EMSA, using as a probe the [*γ*‐^32^P] end‐labeled double‐stranded DNA oligonucleotide (40,000 cpm; 1.29 nm), containing the DNA‐binding site for HNF1A from the *GLUT2* gene promoter. Lane 1 carried free probe. In lanes 2 and 5, no competitor was added. Lanes 2–4 carried rHNF1A. Lanes 5–7 carried rHNF1A Q141fs*47. Lanes 3 and 6 carried 300‐fold molar excess of unlabeled probe as specific competitor (SC). Lanes 4 and 7 carried 300‐fold molar excess of unlabeled *E. histolytica* TATTTAAA oligonucleotide used as unspecific competitor (UC). (B) 12% SDS‐PAGE of protein extracts from [^35^S]‐Methionine‐labeled transcription/translation coupled reactions expressing no recombinant protein (lane 2), rHNF1A protein (lane 3), and HNF1A Q141fs*47 polypeptide (lane 4). Lane 1 contained molecular weight markers. (C) The gel showed in *B* was exposed to an autoradiography film and developed. Lane 2 contained no recombinant protein synthesized. Lane 3 contained rHNF1A polypeptide. Lane 4 contained rHNF1A Q141fs*47 protein.

To evaluate the HNF1A and HNF1A Q141Hfs*47 DNA‐binding capacity, they were synthesized in vitro and assayed in EMSA experiments. Two DNA‐protein complexes were observed with recombinant HNF1A (rHNF1A), demonstrating their DNA‐binding activity (Fig. 2A), where the complex 1 was more abundant than the complex 2 (Fig. 2A, lane 2). We observed that both complexes were specific because a 300 molar excess of unlabeled probe abolished their formation (Fig. 2A, lane 3) and an unrelated DNA sequence did not affect the DNA‐rHNF1A complex formation (Fig. 2A, lane 4). In the case of rHNF1A Q141Hfs*47, two barely visible DNA‐protein complexes were detected with almost similar mobility to that shown by the rHNF1A complex 2 (Fig. 2A, lane 5). The amount of these complexes diminished with unlabeled probe (300 molar excess) (Fig. 2A, lane 6), but it was unaffected with an unrelated competitor (Fig. 2A, lane 7). Thus, the DNA‐binding activity was dramatically reduced because of the c.422_423InsT mutation. To verify the synthesis of proteins in vitro, we labeled them with L‐^35^S‐Methionine. Even though we did not see any difference in protein patterns (Fig. 2B), we detected the ^35^S‐Methionine‐labeled polypeptides of expected sizes (Fig. 2C).

### The HNF1A DNA‐binding activity was abolished by the HNF1A Q141Hfs*47

**Figure 3:**
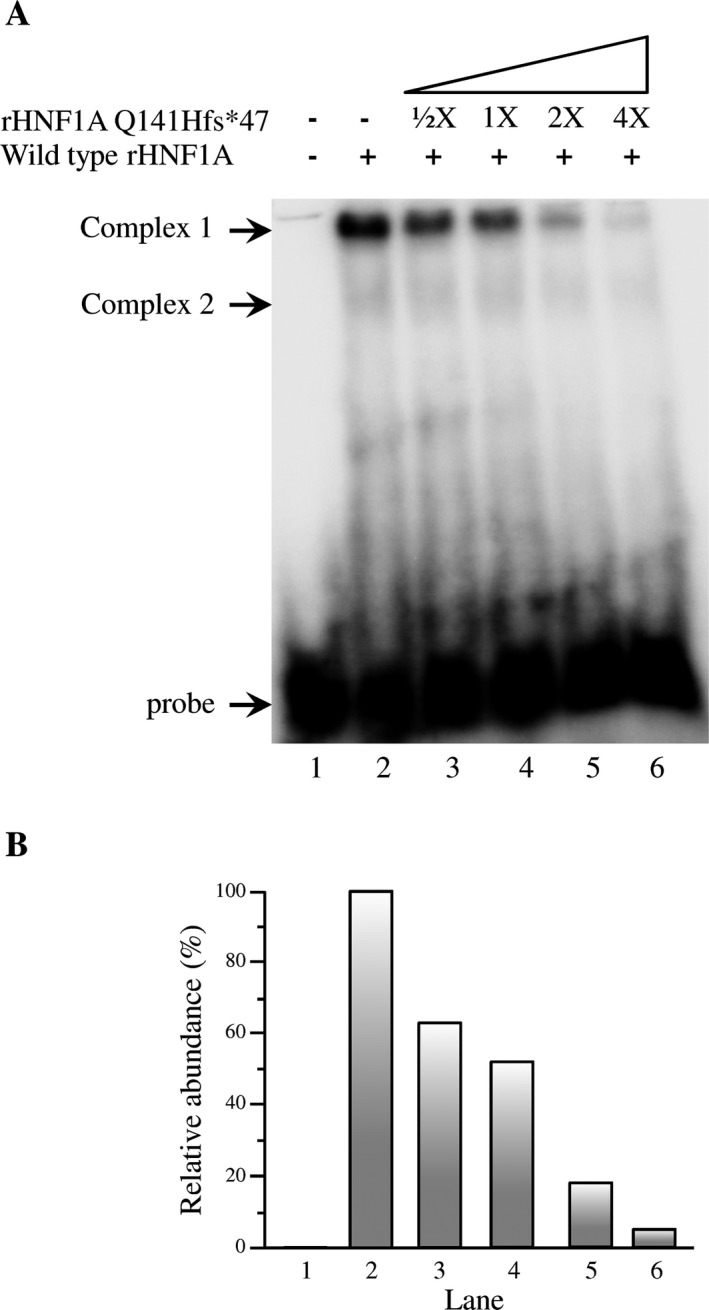
The recombinant HNF1A Q141fs*47 polypeptide exerted a negative effect on the DNA‐binding activity of HNF1A. (A) rHNF1A and rHNF1A Q141fs*47 polypeptides were expressed in vitro in a coupled transcription/translation system as described. Five μL of reaction mixture of rHNF1A polypeptide synthesized in vitro were used in each assay. Then, increasing amounts of the rHNF1A Q141fs*47 polypeptide were added (lanes 3 to 6) and assayed by EMSA, using as a probe the [*γ*‐^32^P] end‐labeled double stranded DNA oligonucleotide (40,000 cpm; 0.52 nm) containing the DNA‐binding site for HNF1A from the *GLUT2* gene promoter. Lane 1 contained free probe. In lane 2, no mutant polypeptide was added; Lanes 3–6 contained 2.5, 5, 10, and 20 μL, respectively, of reaction mixture containing the rHNF1A Q141fs*47 polypeptide. (B) Radioactivity from EMSA assays was determined as described in Methods. We considered the amount of complex one shown in lane 2 as 100%.

Outstandingly, the HNF1A DD was unaffected by the c.422_423InsT mutation (Fig. 1). Therefore, we determined that the HNF1A Q141Hfs*47 could exert a negative effect on the HNF1A DNA‐binding capacity by the formation of non‐functional heterodimers. To test this assumption, more EMSA experiments were performed using increasing quantities of rHNF1A Q141Hfs*47, while keeping fixed the quantity of rHNF1A (Fig. 3). A remarkably negative effect of the mutant polypeptide over the rHNF1A DNA‐binding activity was observed, clearly affecting the complex 1 formation, which was proportional to the quantities of rHNF1A Q141Hfs*47 used (Fig. 3A). We also observed a fourfold excess of rHNF1A Q141Hfs*47 that diminished the complex 1 formation in nearly 95% (Fig. 3, lane 6, A and B).

### Intracellular localization of EGFP‐HNF1A and EGFP‐HNF1A Q141Hfs*47 fusion proteins

**Figure 4:**
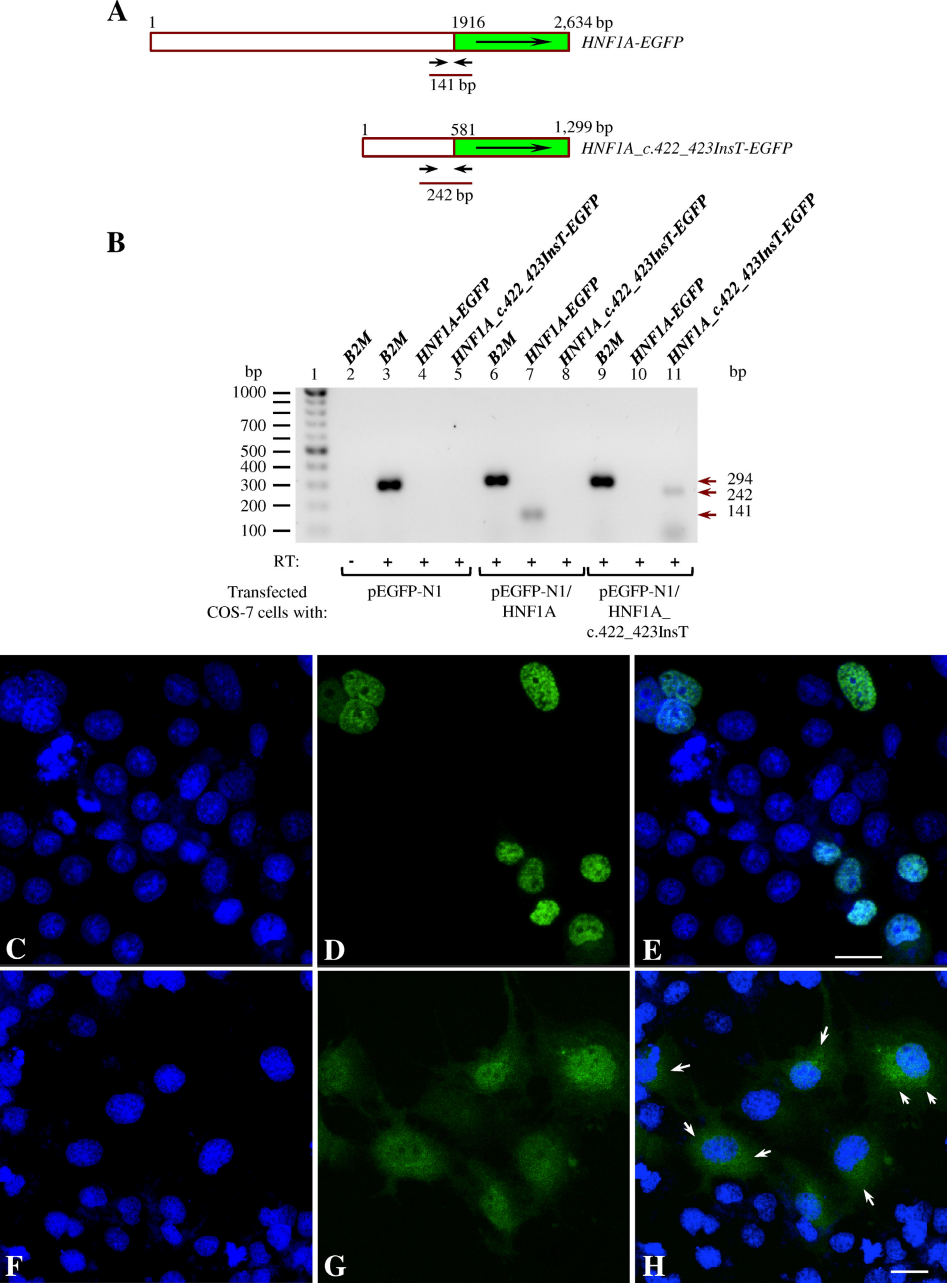
The HNF1A Q141Hfs*47 mutant polypeptide is inefficiently translocated to the nucleus. (A) Diagram of the *HNF1A‐EGFP* and *HNF1A c.422_423InsT‐EGFP* fusion genes showing the location of primers used in expression assays by RT‐PCR. (B) RT‐PCR products analyzed by electrophoresis in a 1% agarose gel stained with ethidium bromide. Lane 1 contained molecular size markers. Lanes 2–5 contained RT‐PCRs performed with RNA isolated from COS‐7 cells transfected with the pEGFP‐N1 plasmid. Lanes 6–8 contained pEGFP‐N1/HNF1A. Lanes 9–11 contained pEGFP‐N1/HNF1A_c.422_423InsT. Lanes 2, 3, 6, and 9 contained RT‐PCR amplifications for *B2M* (*β*‐2‐microglobulin). Lanes 4, 7, and 10 contained *HNF1A‐EGFP*. Lanes 5, 8, and 11 contained *HNF1A_c.422_423InsT‐EGFP*. RT, reverse transcriptase. (C–H) confocal microscopy of COS‐7 cells transfected with plasmid pEGFP‐N1/HNF1A (C–E) or pEGFP‐N1/HNF1A_c.422_423InsT (F–H). Nuclei stained with Hoechst 33342 (C, F, blue channel). Fluorescence of EGFP fusion proteins (D, G, green channel). (E) Merge of fluorescence signals of C and D. (H) Merge of fluorescence signals of F and H. Scale bar, 30 μm.

The plasmids pEGFP‐N1/HNF1A and pEGFP‐N1/HNF1A_c.422_423InsT were transiently transfected into COS‐7 cells. The expression of recombinant mRNAs was first verified by RT‐PCR (Fig. 4A,B). The confocal microscopy revealed that EGFP‐HNF1A was localized only inside the nuclei of transfected cells (Fig. 4C–E) just as expected. However, although the EGFP‐HNF1A Q141Hfs*47 was localized inside nuclei as well, it was also found in cytoplasm (Fig. 4F–H, arrows), supporting our initial assumption of a defect in the translocation toward the nuclei.

### Comparative structural analysis of HNF1A and HNF1A Q141Hfs*47

To have a molecular explanation of the effect caused by the insertion, we performed a homology modeling of HNF1A Q141Hfs*47 (residues 87–164), using as a template the HNF1A (87–164 residues) bound to a 20 bp DNA fragment (PDB‐ID 1IC8). The model showed the following values: ModPipe Quality Score of 1.29645; TSV Mod NO35 of 0.813; GA341 of 1.00; E‐value of 0; z‐DOPE of −0.92. They indicated a good quality model. In addition, the model was submitted to evaluation in SAVES web page. The PROCHECK program indicated that the protein parameters are within good quality values (data not shown). The Ramachandran plot for mutant polypeptide model displayed 93.8% of total residues in the most favored regions and 6.2% in the additional allowed regions, also indicating a good quality. The ERRAT2 program indicated that the overall quality factor for HNF1A Q141Hfs*47model was 94.286, which is close to the 95% value obtained with good high‐resolution structures. The PROVE program results were: the *Z*‐score mean = −0.638, the *Z*‐score standard deviation = 1.506, and the *Z*‐score RMS = 1.631 at a resolution of 2.00 Angstroms.

**Figure 5:**
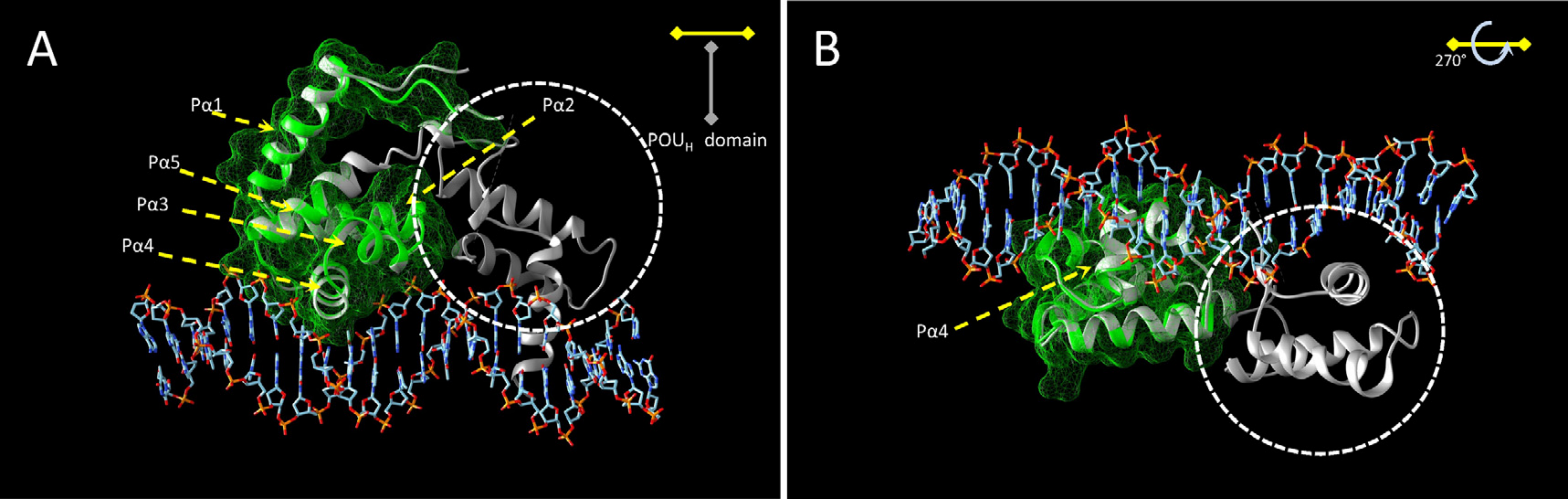
Structural comparison of the molecular models of HNF1A Q141fs*47 and HNF1A polypeptides. The molecular structure of the HNF1A Q141Hfs*47 polypeptide was theoretically determined with the Modeller program using as a template the structure of the HNF1A bound to DNA (PDB ID 1IC8). Both structures were superimposed with the Chimera program. The mutant HNF1A Q141Hfs*47 polypeptide is displayed as green ribbons and with its surface as a green mesh. The normal HNF1A protein is shown as gray ribbons. (A) The *α*‐helixes P*α*1 to P*α*5 of the POU_S_ domain are indicated with arrows. Encircled is the POU_H_ domain of the HNF1A polypeptide. Only the P*α*4 helix involved in DNA‐binding activity is shown. (B) Structures shown in A were rotated 270° about the horizontal axis.

The comparison between HNF1A and HNF1A Q141Hfs*47 structures was performed with the Match Maker module of Chimera. Only one HNF1A chain (bound to DNA) is displayed for structure comparison (Fig. 5). There was almost a perfect match between the normal and the mutant backbones (Fig. 5, gray and green chains, respectively). The five *α*‐helixes of the HNF1A POU_S_ domain (16) were present, but the P*α*5 helix was truncated (16 residues in the mutant, 26 residues in the normal) (Figs. 1, 5). The P*α*4 was located within one major groove in both proteins, and was responsible for most of the interactions between the HNF1A POU_S_ domain (residues 140–149) and DNA (Figs. 1, 5). Figure 5 reveals that the HNF1A Q141Hfs*47 also lacks in POU_H_ domain (encircled in Fig. 5A), domain that is essential for binding to the other major groove of DNA (Fig. 5A,B).

**Figure 6:**
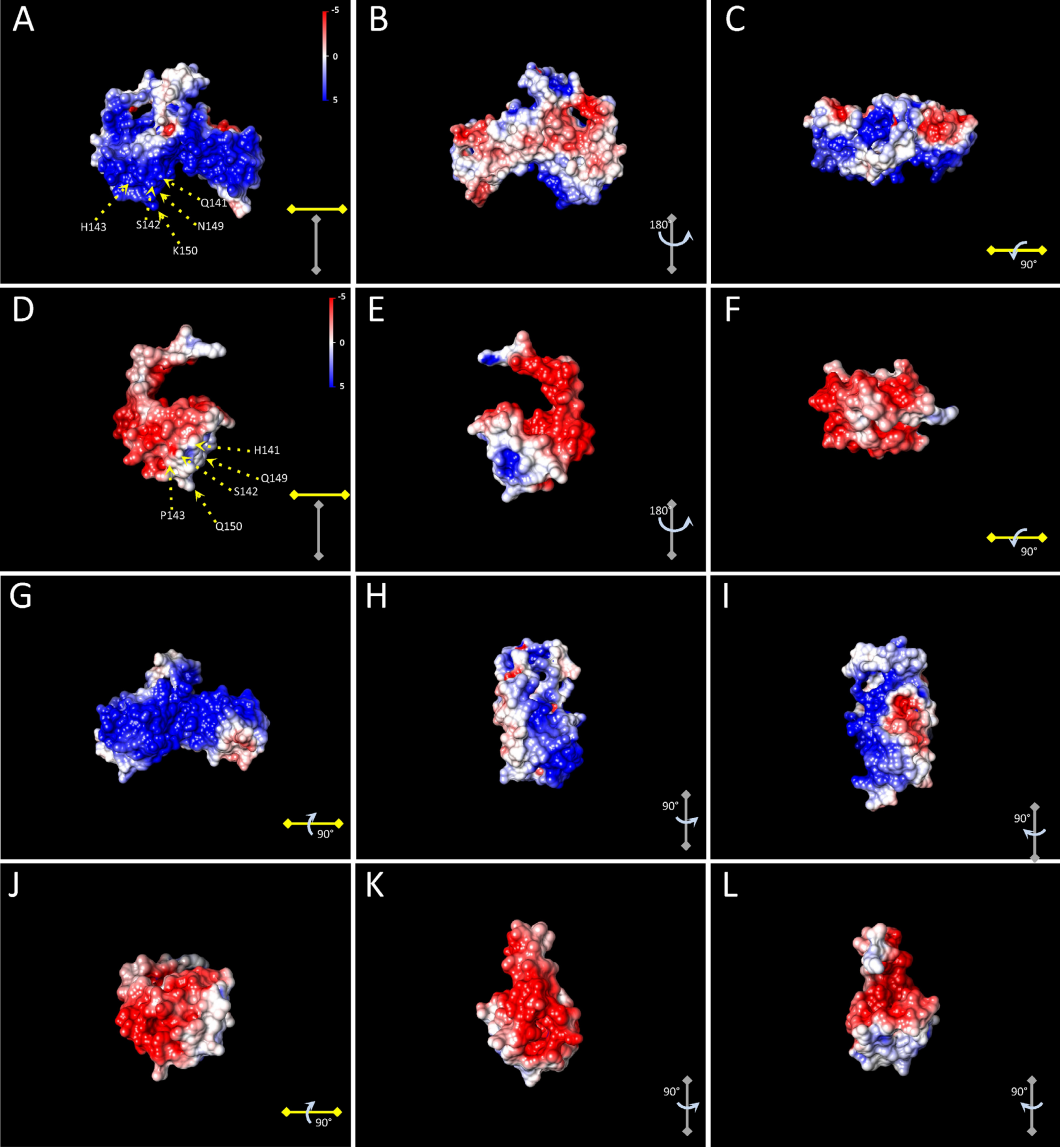
Comparison of the surface electrostatic potential of HNF1A and HNF1A Q141Hfs*47 polypeptides. The electrostatic potential for each molecule was calculated as described in Methods. Different faces of the normal HNF1A polypeptide are shown in A, B, C, G, H, and I. Different faces of the HNF1AQ141Hfs*47 polypeptide are shown in D, E, F, J, K, and L. Residues located in the POU_S_ domain of wt HNF‐1*α* polypeptide involved in interactions with DNA and pointed by arrows are shown in *A*. Mutated residues located in the POU_S_ domain from the HNF1A Q141Hfs*47 polypeptide are shown in D.

To determine the EP values of HNF1A and HNF1A Q141Hfs*47 molecular models, we first converted the PDB files into PQR format using the PDB2PQR server. Then, the EP was determined with the Adaptive Poisson‐Boltzmann Solver (APBS) software, and visualized with Chimera. The EP values were different in the HNF1A and HNF1A Q141Hfs*47 proteins (Fig. 6). The HNF1A protein had a positive charge on most of the surface (Fig. 6A). On the contrary, the HNF1A Q141Hfs*47 had a negative value (Fig. 6D). Outstandingly, most of the residues involved in the DNA‐protein interactions changed in mutant polypeptides and consequently, the EP on the surface of these residues were slightly positive, tending to neutrality (Fig. 6A,D,G,J). Other differences were observed in other surface regions of molecules that might have a negative effect on interactions with other proteins (Fig. 6).

**Figure 7:**
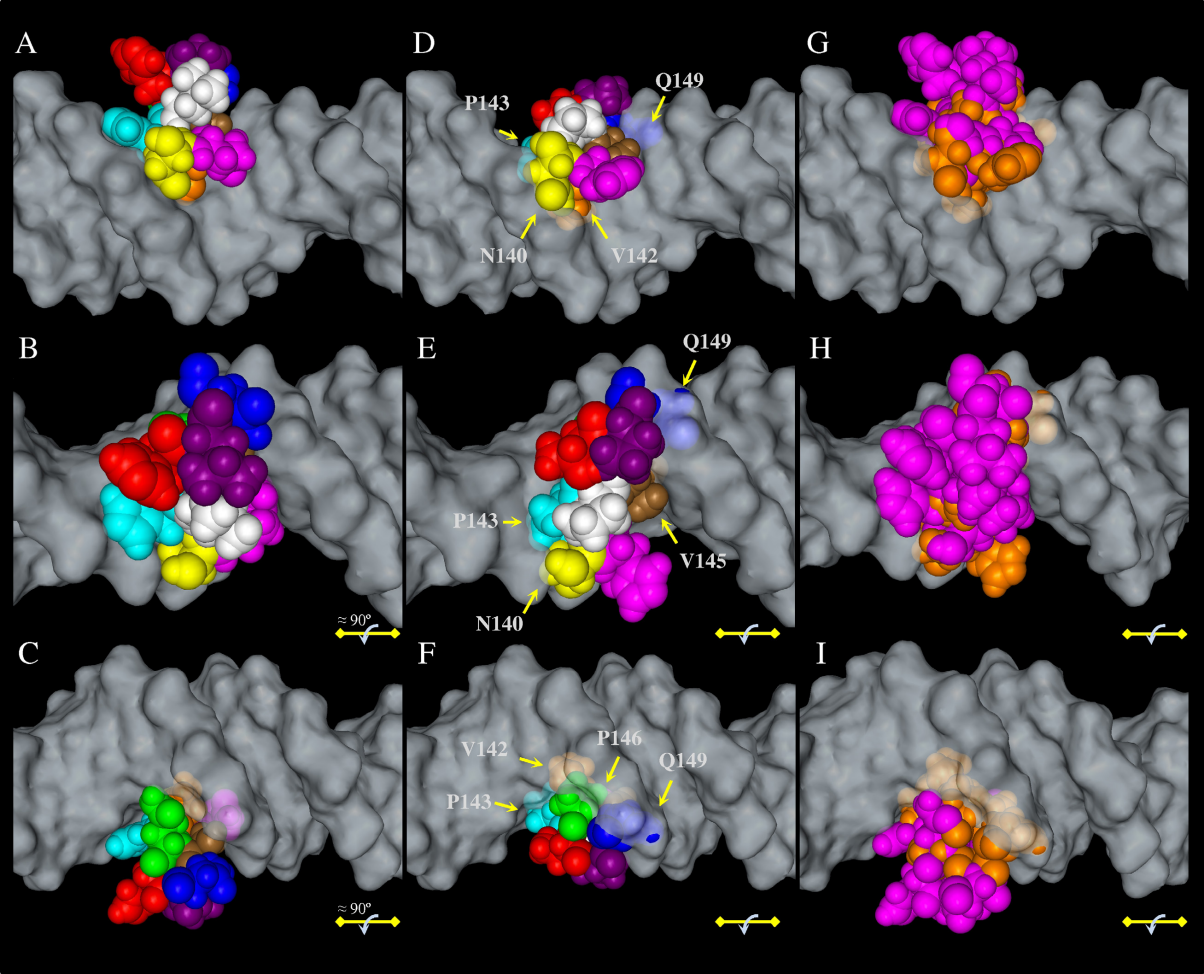
Structural comparison of the DNA–protein interface of the POU_S_ domains in HNF1A and HNF1A Q141Hfs*47 polypeptides. (A–C) Residues of normal HNF1A that contacted the major groove of DNA (N140, Q141, S142, H143, Q146, and N149), as well as those that did not (L144, S145, H147, and L148). (D–F) Residues of HNF1A Q141Hfs*47 protein that contacted DNA (N140, H141, V142, P143, P146, and Q149), and those that did not (P144, V145, T147, and P148). The color codes for HNF1A and HNF1A Q141Hfs*47 polypeptides were: N140 – yellow for both proteins; Q141 and H142 – magenta; S142 and V142 – orange; H143 and P143 – cyan; L144 and P144 – white; S145 and V145 – brown; Q146 and P146 – green; H147 and T147 – red; L148 and P148 – purple; N149 and Q149 – blue. Molecule rotated nearly 90° (B, E, H) and 180° (C, F, I) along the DNA alpha helix. (G–I) Superimposed HNF1A and HNF1A Q141Hfs*47 POU_S_ domain structures. HNF1A – cyan; HNF1A Q141Hfs*47 – green.

The analysis of the DNA–protein interface of the HNF1A POU_S_ domain with MMV showed residues making direct contact with DNA (N140, Q141, S142, H143, Q146, and N149), as well as others that did not have contact with DNA (L144, S145, H147, and L148) (Figs. 1, 7A–D). When this molecule was rotated approximately 90° (Fig. 7B) and 180° (Fig. 7C) along the DNA alpha helix, a perfect assembly of the residues 140–149 in the DNA major groove was observed. However, the analysis of the HNF1A Q141Hfs*47 model revealed problems in the stereochemistry of some residues (Fig. 7E,F, arrows). Steric clashes were easily seen for residues N140, V142, P143, V145, P146, and Q149 (Fig. 7D–F). Finally, we superimposed both structures to contrast their differences (Fig. 7G–I). These modeling evidences helped to understand the lack of DNA‐binding capacity of the HNF1A Q141Hfs*47 polypeptide (Fig. 2).

## Discussion

Mutations in the *HNF1A* gene are characterized by a progressive deficiency in glucose‐stimulated insulin secretion. The functional analysis of mutations in MODY 3 is useful to identify the corresponding molecular defects that might contribute to unveil the mechanism of *β*‐cell impairment in these patients (Galán et al. 2011). The HNF1A protein is a transcription factor involved in the expression of *β*‐cell genes like pyruvate kinase, GLUT2, and insulin. The HNF1A protein is synthesized in the cytoplasm and then imported to the nucleus where it binds as homodimer or heterodimer, to a specific cis‐acting sequence located in promoters of target genes, which allows the interaction with the basal transcription machinery and other tissue‐specific factors to stimulate transcription (Maston et al. 2006). There has been reports of a close relationship between the type and position of mutations in the *HNF1A* gene with the age at diagnosis and severity of diabetes phenotype; therefore, affecting diagnose and treatment given to patients with MODY (Bellanné‐Chantelot et al. 2008). Moreover, these patients could be incorrectly treated as type 1 diabetic patients. Patients with MODY 3 are sensitive to low doses of sulfonylureas and usually do not require insulin therapy during at least 6 months after the diabetes diagnose (Slingerland 2006).

In this work, we screened mutations in the *HNF1A* gene in patients with T2D and compared them against non‐diabetic controls; and found two polymorphisms in both (type 2 diabetic patients and controls). The I27L polymorphism is located within the dimerization domain of the HNF1A polypeptide. I27 is a conserved residue among several species, which suggests that it has a potential functional role in HNF1A (Ryffel 2001). An association between the I27L polymorphism with an increased risk of T2D has been observed in a case–control study performed in Swedish population (Holmkvist et al. 2006), which was supported by in vitro findings indicating that the L allele was associated with a decreased transcriptional activity in HeLa and INS‐1 cells. Other study showed that the I27 allele of the *HNF1A* gene was inversely associated with HDL serum levels. These data suggest that the *HNF1A* gene may be involved in the regulation of HDL‐c levels in serum and that the I27L allele could be used as a risk marker for atherosclerosis (Babaya et al. 2003). We found that the I27L polymorphism frequency was high in both diabetic and control groups, although there were no significant differences in the HDL concentration in these groups (Pinés Corrales et al. 2010). We believe that this polymorphism may be ancestry informative marker in the population studied, as it was in the case of R230C in the *ABCA1* gene (Acuña‐Alonzo et al. 2010).

Mutations in MODY3 are known to affect one or more of the HNF1A properties, including dimerization, DNA‐binding, and transactivation (Chi et al. 2002). The *HNF1A c.422_423insT* mutation was located within the DBD, specifically in the P*α*4 *α*‐helix of the POU_S_ domain (Figs. 1, 5).We found a PolyPhen‐2 score of 1.0 for HNF1A Q141Hfs*47, which suggests a severe negative effect on its functionality (Adzhubei et al. 2010, 2013; Wei et al. 2011). The HNF1A Q141 is a highly conserved residue in species and the POU‐contains proteins such as Pit1, Oct‐1, and Oct‐2. The Q141 and Q130 residues establish a network of hydrogen bonds with DNA; therefore, the Q141 has a key role in DNA‐binding activity (Chi et al. 2002). The EMSA results indicated a very low DNA‐binding capacity of rHNF1A Q141Hfs*47 when assayed with the GLUT2 promoter probe, which was effectively bound by the HNF1A protein (Fig. 2). As the dimerization domain of the recombinant HNF1A Q141Hfs*47 mutant polypeptide was identical to that of the normal HNF1A polypeptide, it was expected that the dimerization capacity would remain unchanged. The EMSA experiments (Fig. 3) suggested a dominant‐negative effect of the mutant polypeptide. Thus, the truncated protein might bind HNF1A forming a non‐functional dimer lacking in DNA‐binding activity. However, more studies are needed to demonstrate this hypothesis. Other studies have shown that the P291fsinsC polymorphism causes the production of a truncated polypeptide that retains the dimerization capacity and the DNA‐binding activity, but lacks in the transactivation domain; it has a dominant negative effect due the nonfunctional dimer formation between wild‐type HNF1A and P291fsinsC mutant polypeptides showing no capacity to activate target genes (Yamagata et al. 1998). This can occur when the gene product forms active complexes with itself (and possibly with other proteins). Mutant HNF1A with an intact dimerization domain, are suggested to impair pancreatic *β*–cell functions in a dominant negative manner by forming nonproductive dimers with the normal protein (Wang et al. 1998). In nonmutant HNF1A, the DCoH binds to HNF1A dimers to stabilize it, resulting in an increased transcription activity in vivo (Mendel et al. 1991; Wan et al. 2005); therefore, the binding between the mutant HNF1A protein and DCoH (dimerization cofactor of HNF1/pterin‐4alpha‐carbinolamine dehydratase) can be unstable.

In conclusion, the etiology of diabetes in patient D16 may be caused by the dominant negative effect of the HNF1A Q141Hfs*47 mutant polypeptide, consequently affecting the expression level of many HNF1A‐dependent genes involved in glucose metabolism and the normal beta cell function. Therefore, it is necessary to perform other functional analysis of variations in the *HNF1A* gene to understand the great diversity of phenotypes observed in patients and families with *HNF1A* mutations. The improvement of the genetic analysis of MODY 3 is of outmost importance, since upon diagnosis, several patients can be switched to sulfonylureas thus improving their compliance, preventing microvascular complications and most importantly ameliorating their quality of life.

## Conflict of Interest

The authors declare to have no conflicts of interest.
